# Upregulated influenza A viral entry factors and enhanced interferon-alpha response in the nasal epithelium of pregnant rats

**DOI:** 10.1016/j.heliyon.2022.e09407

**Published:** 2022-05-11

**Authors:** Tusar Giri, Santosh Panda, Jeannie C. Kelly, Carlo Pancaro, Arvind Palanisamy

**Affiliations:** a Department of Anesthesiology, Washington University School of Medicine, St. Louis, Missouri, USA; b Department of Pathology, Washington University School of Medicine, St. Louis, Missouri, USA; c Department of Obstetrics and Gynecology, Washington University School of Medicine, St. Louis, Missouri, USA; d Department of Anesthesiology, University of Michigan Medical School, Ann Arbor, Michigan, USA

**Keywords:** Influenza A, Host-pathogen interaction, Nasal epithelium, Pregnancy, TLR7, pDC

## Abstract

Despite the increased severity of influenza A infection in pregnancy, knowledge about the expression of cell entry factors for influenza A virus (IAV) and the innate immune response in the nasal epithelium, the primary portal of viral entry, is limited. Here, we compared the expression of IAV cell entry factors and the status of the innate immune response in the nasal epithelium of pregnant *vs*. non-pregnant female rats. IAV cell entry factors — sialic acid [SA] α-2,3- and α-2,6-linked glycans for avian and human IAV, respectively — were detected and quantified with lectin-based immunoblotting and flow cytometry. Baseline frequencies of innate immune cell phenotypes in single cell suspensions of the nasal epithelium were studied with flow cytometry. Subsequently, the magnitude of interferon and cytokine responses was studied with ELISA and cytokine arrays after intranasal resiquimod, a Toll-like receptor 7/8 agonist that mimics IAV infection. We noted substantially increased expression of cell entry factors for both avian and human IAV in the nasal epithelium during pregnancy. Assessment of the innate immune state of the nasal epithelium during pregnancy revealed two previously unreported features: (i) increased presence of tissue-resident plasmacytoid dendritic cells, and (ii) markedly enhanced release of interferon-α but not of the other interferons or cytokines 2 h after intranasal resiquimod. Collectively, our findings challenge the conventional notion of pregnancy-induced immunosuppression as a cause for severe influenza A disease and suggest the need for focused studies on viral tropism during pregnancy to better understand the proximate cause for the observed immunopathology.

## Graphical abstract

**Figure:**
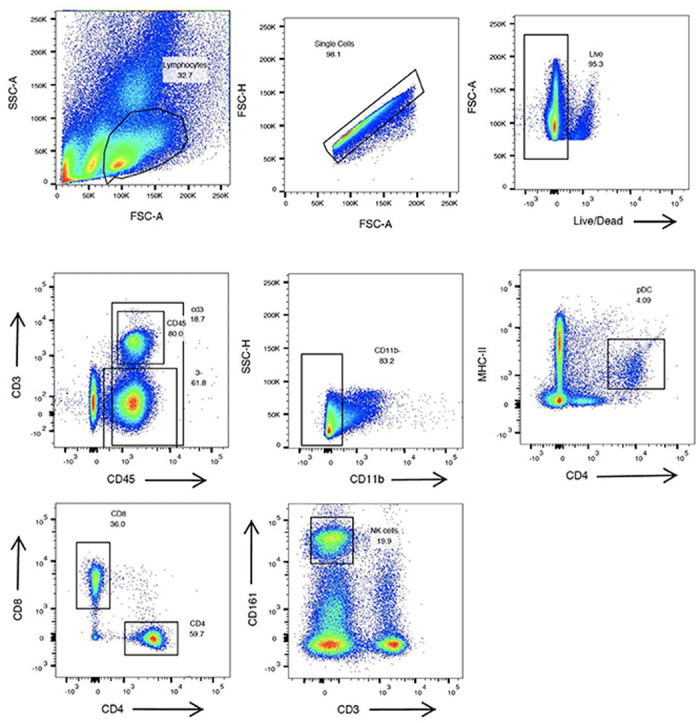

**Importance**

Pregnant women have borne the brunt of influenza A pandemics with high rates of severe disease and death, often attributed to systemic immunosuppression and the cardio-respiratory changes associated with pregnancy. However, a detailed characterization of how the influenza A virus interacts with the pregnant host has been lacking. In a preclinical model, we show for the first time that pregnancy is characterized by a significant increase in factors necessary for the cellular entry of influenza A virus and a robust innate immune response with exaggerated release of interferon-α after chemical provocation of the nasal epithelium. Our preclinical data underlines the critical importance of comprehensively investigating mechanisms of host-pathogen interaction in the nasal epithelium of pregnant human subjects. An understanding that pregnant women are not only at risk for severe complications but are more likely to get infected with influenza A virus could be a significant motivator to seek vaccination.

## 1 Introduction

Pregnancy is a unique but poorly understood immunological state characterized by the need to tolerate an allogeneic fetus *vs*. the ability to defend against invasive microbes [1, 2, 3, 4, 5, 6]. These systemic immune changes are nuanced and involve gestational age-specific adaptation and fine tuning [7, 8, 9, 10, 11, 12, 13]. Along with physiological changes in the respiratory tract, pregnant women are vulnerable to severe respiratory viral infections [14, 15]. This is epitomized by mortality rates of approximately 15–30% in pregnant women during the previous influenza A pandemics [16, 17]. In the most recent 2009 H1N1 pandemic, approximately 5% of all deaths were among pregnant women despite accounting for only 1% of all infections [18].

Despite the increased severity of pandemic and seasonal influenza A in pregnant women, foundational aspects of the interaction between the influenza A virus (IAV) and the pregnant host remain poorly characterized. This knowledge void is particularly striking at the nasal epithelium, the primary portal of viral entry and the foremost site for innate immune defense in the host. For the cellular entry of IAV, sialylated moieties linked to galactose (sialoglycans) in the airway epithelial cells are necessary [19]. Though both avian and human IAV entry receptors (α-2, 3- and α-2,6-linked sialylated glycans, respectively) are expressed in the nasal epithelium in humans [19], the question whether their expression is modulated by pregnancy remains unresolved. Because pregnancy is a state of increased systemic sialylation presumably from the effect of pregnancy-associated hormones [20, 21, 22, 23, 24], we hypothesized that this would result in sialylation of IAV cell entry glycans in the nasal epithelium. Furthermore, the extent and magnitude of antiviral responses in the nasal epithelium, especially type I interferon (IFN–I) release and signaling mediated by the Toll-like receptor (TLR) pathway, remains poorly studied during pregnancy. Because pregnancy is associated with reduced IFN-1 response to IAV subtype H1N1 infection in cultured peripheral blood mononuclear cells [25], we hypothesized that pregnancy would be characterized by a deficient IFN-1 response in the nasal epithelium.

## 2 Results

**Figure 1:**
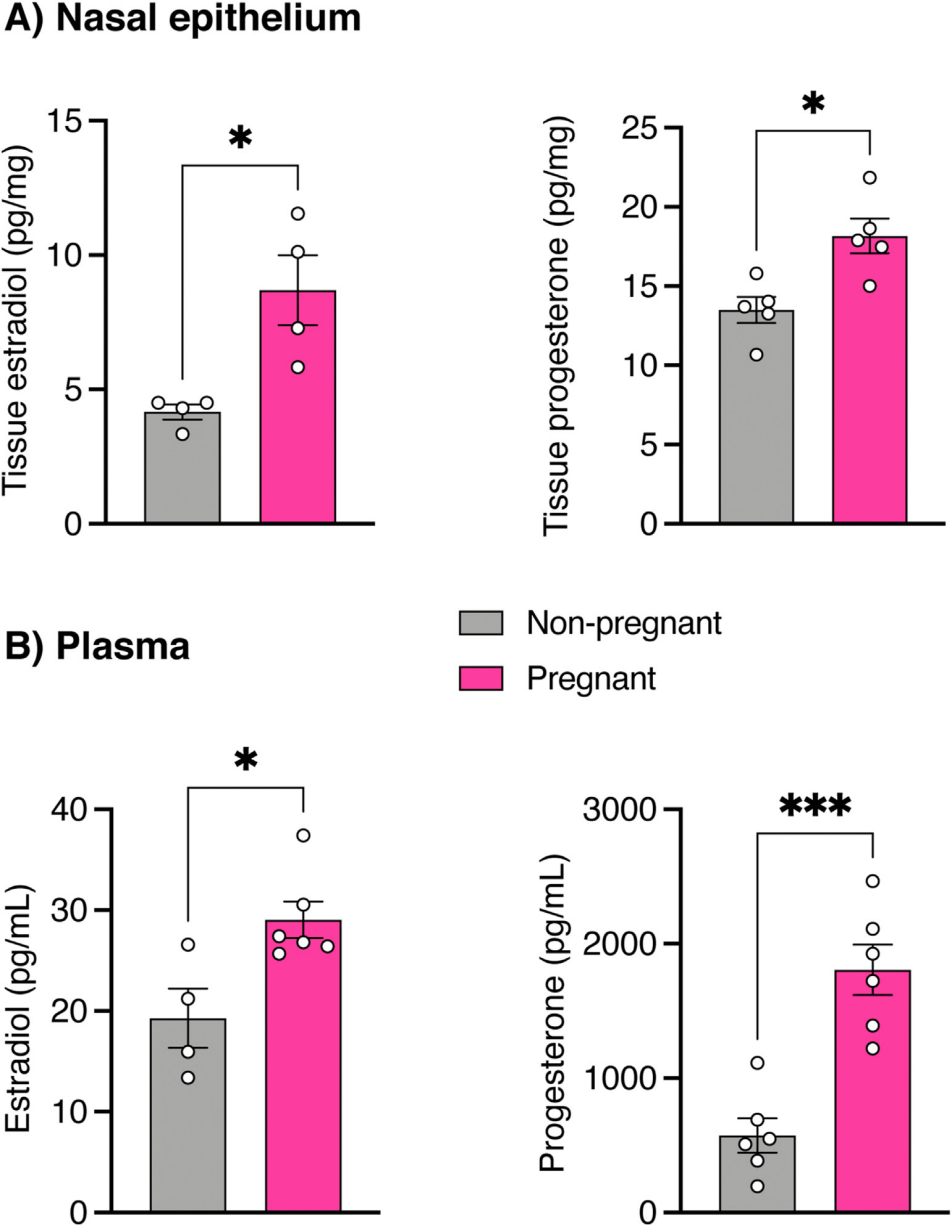
Concentration of pregnancy-associated hormones in the nasal epithelium and plasma. Scatter plots showing the concentration of estradiol and progesterone in the nasal epithelium (A) and plasma (B). The markedly elevated concentration of estradiol and progesterone in the nasal epithelium during pregnancy mirrored the increase in plasma concentration of these hormones. Data were analyzed with Welch's t-test and presented as mean ± SEM (n = 4–6 per condition); ∗p ≤ 0.05, ∗∗∗p ≤ 0.001.

We first assessed whether the hormonal changes of pregnancy were reflected at the nasal epithelial tissue. Tissue concentration of both 17-β estradiol and progesterone were substantially higher in the nasal epithelium of pregnant compared to non-pregnant rats (Figure 1a). This increase reflected the elevated plasma hormonal levels associated with pregnancy (Figure 1b).

**Figure 2:**
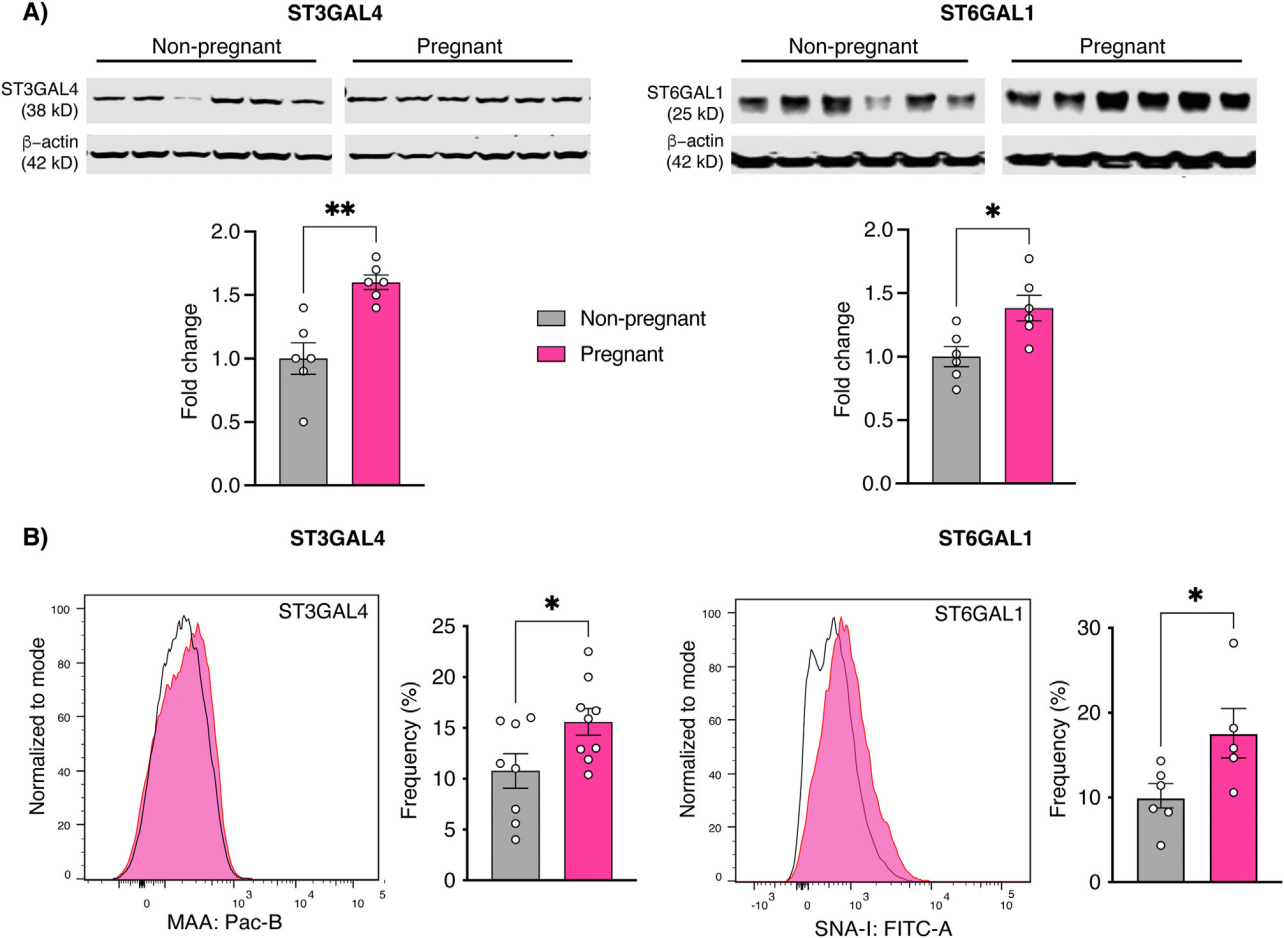
Increased expression of influenza A viral receptors in the nasal epithelium of pregnant rats. (A) Immunoblots for ST3GAL4 and ST6GAL1 and their accompanying densitometric quantification presented as scatter plots below. Pregnancy was associated with an increase in both ST3GAL4 and ST6GAL1 expression, suggesting increased availability of sialyltransferases for the synthesis of sialic acid [SA] α-2,3, and α-2,6-linked glycans, respectively, for IAV entry. β-actin and rat intestine were used as loading and positive controls, respectively. Immunoblots were cropped to arrange non-pregnant samples before pregnant samples to ensure consistency with data presentation similar to other figures. Uncropped immunoblot images are provided in the Supplementary File. (B) Representative flow cytometry histograms depicting the increased expression of Maackia amurensis agglutinin (MAA; ST3GAL4) and Sambucus nigra agglutinin-I (SNA-I; ST6GAL1) on the surface of nasal epithelial cells of pregnant *vs*. age matched non-pregnant rats, along with their respective frequency scatter plots. Black-line histograms represent MAA and SNA-I expression on the nasal epithelial cells of non-pregnant rats while the shaded histograms represent data from pregnant rats. Data were pooled from three (MAA; n = 3 each) and two (SNA-I; n = 3 each) independent experiments and analyzed with Welch's t-test (mean ± SEM; ∗p ≤ 0.05, ∗∗p ≤ 0.01).

Both ST3GAL4 and ST6GAL1, representing the sialyltransferase enzymes that mediate synthesis of α-2,3- and α-2,6-linked sialylated glycans, respectively, were markedly increased in immunoblots of nasal epithelial lysates from pregnant *vs*. non-pregnant rats (Figure 2a). To confirm that the increase of these IAV entry factors was due to increased expression in epithelial cells, we performed lectin-based flow cytometry in single cell suspensions generated from freshly dissected nasal epithelial tissue (Figure 2b). Consistent with our immunoblotting results, both cell entry factors were significantly increased in nasal epithelial cells from pregnant samples.

**Figure 3:**
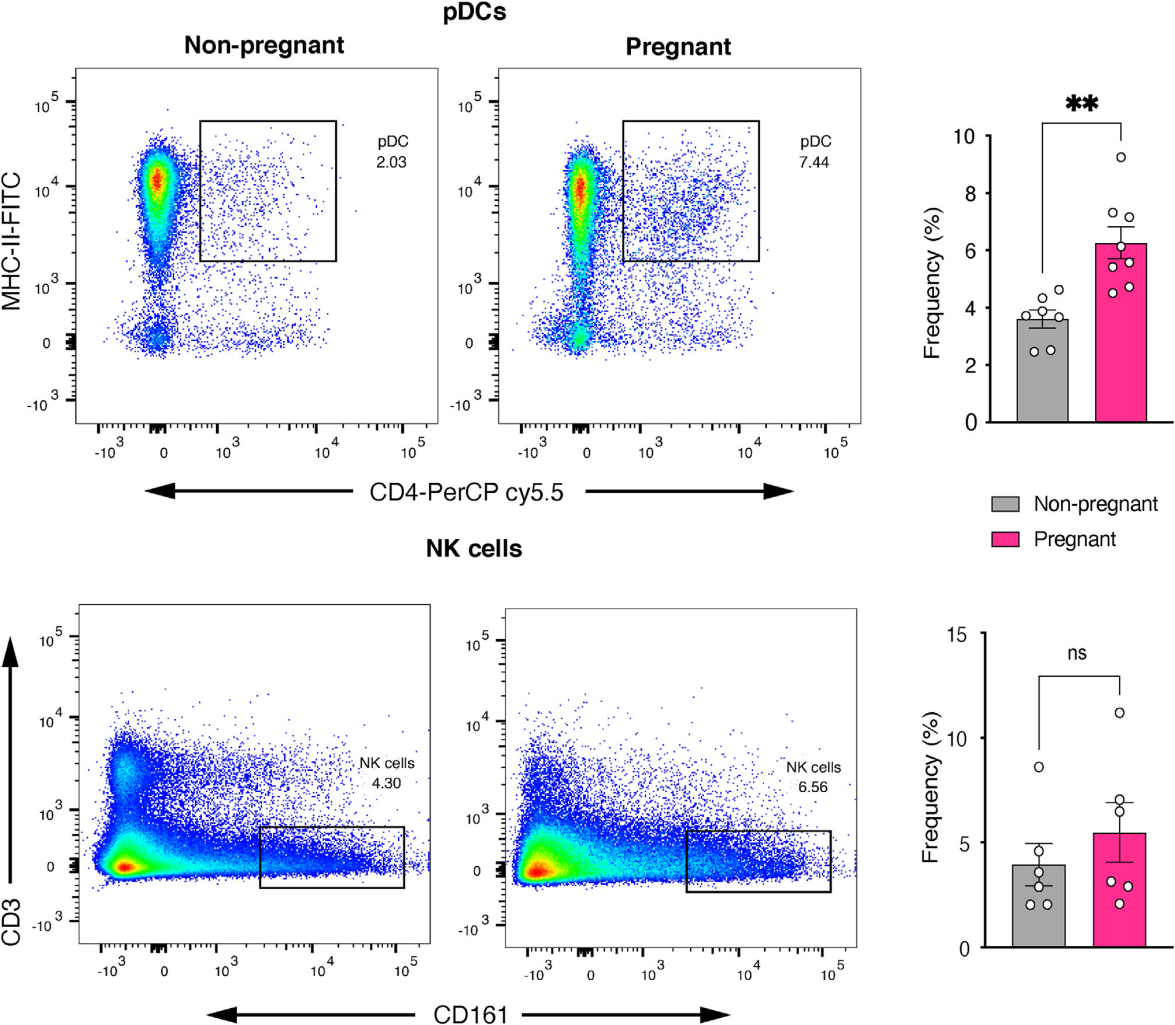
Pregnancy is characterized by increased expression of pDCs in the nasal epithelium. *Upper panel:* Representative FACS two-parameter pseudocolor dot plot depicting the presence of pDCs on the nasal epithelium of non-pregnant and pregnant rats. The area marked with square represent the percentages of pDCs. Scatter plot to the right shows a significant increase in the frequencies of pDCs in the nasal epithelium of pregnant rats. *Lower panel:* Representative FACS two-parameter pseudocolor dot plot depicting the presence of NK cells. Scatter plot to the right shows the frequencies of NK cells in the two groups. Data were pooled from three (pDC; n = 3 each) or two (NK cells; n = 3 each) independent experiments and analyzed with Welch's t-test (mean ± SEM; ∗∗p ≤ 0.01, ns: not significant).

Subsequently, based on our previous observations of upregulated innate immune gene expression in the nasal epithelium of pregnant rats [26], we wanted to examine whether there were any differences in innate immune cell populations between the groups. We evaluated the frequencies of plasmacytoid dendritic cells (pDC) and natural killer (NK) cells in nasal single cell suspensions using flow cytometry. Only pDCs were substantially increased in the pregnant samples, whereas NK cell frequencies were broadly comparable to those in non-pregnant samples (Figure 3).

**Figure 4:**
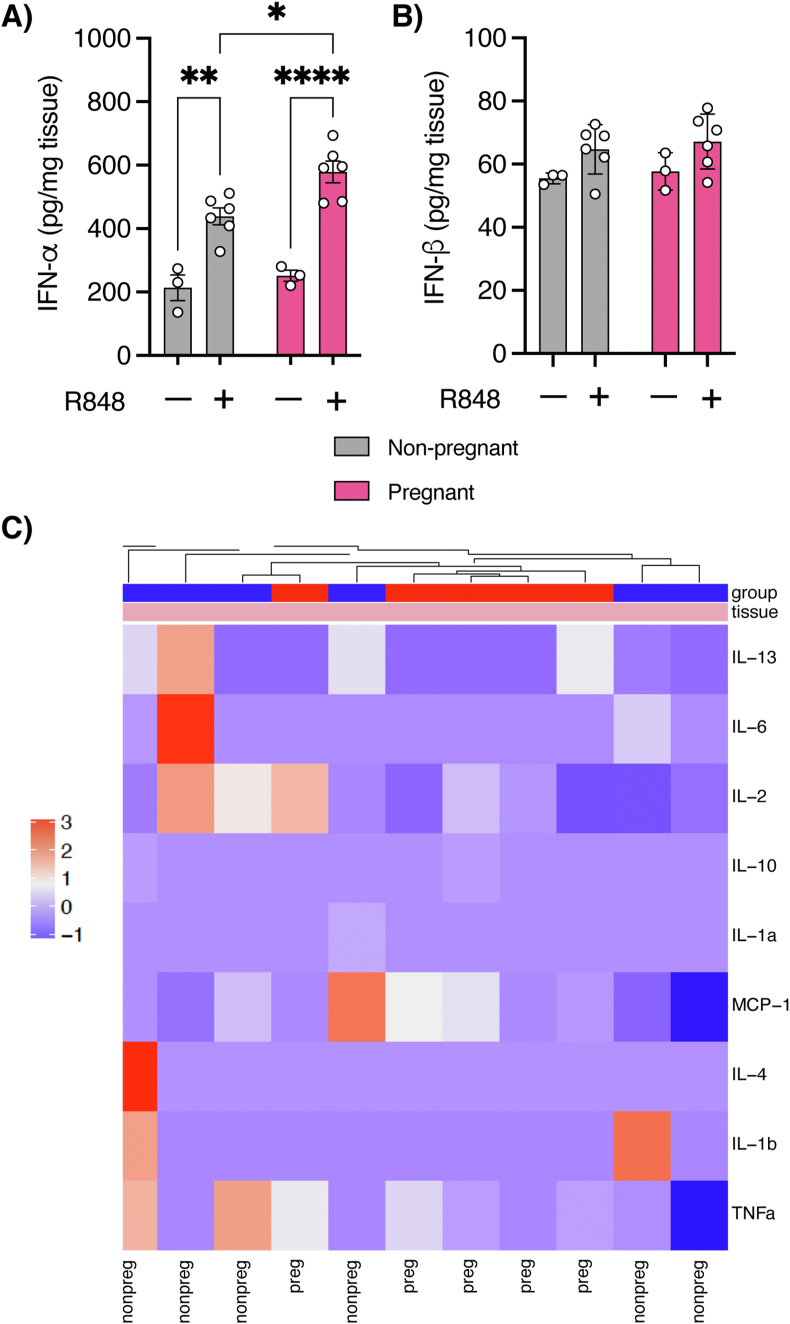
TLR-7 stimulation is associated with robust interferon-α response in the nasal epithelium. Compared to vehicle-only controls, resiquimod was associated with a substantial increase in IFN-α in both pregnant and non-pregnant female rats, confirming stimulation of the TLR-7 pathway. Scatter plots showing significantly increased IFN-α (Α) in the nasal epithelium of pregnant rats 2 h after intranasal resiquimod stimulation, but not IFN-β (Β). For the cytokine experiments (C), the scaled and centered data were plotted as a heatmap in which the different colors represent cytokine expression levels. IFN-γ was excluded from the heatmap analysis because of zero variance, and the other 9 cytokines were subjected to hierarchical clustering by Euclidean distance. There were no significant differences in cytokine expression levels between the two groups. Data were analyzed with either 2-way ANOVA or Wilcoxon-Mann-Whitney test and presented as mean ± SEM (n = 3–6 each); ∗p ≤ 0.05, ∗∗p ≤ 0.01, ∗∗∗∗p ≤ 0.0001.

Motivated by these findings, we sought to examine if the innate immune response in the nasal epithelium was qualitatively different in pregnancy by performing interferon and cytokine expression studies after intranasal instillation of resiquimod, a TLR-7/8 agonist. Two hours after intranasal resiquimod, release of IFN-α was markedly enhanced in pregnant *vs*. non-pregnant rats (Figure 4a). However, we did not observe any differences in the concentration of IFN-β (Figure 4b) or any of the other inflammatory cytokines between the groups (Table 1 and Figure 4c).

**Table 1:** Cytokine response after intranasal resiquimod.

Biomarker	Non-pregnant (pg/mL)	Pregnant (pg/mL)	Fold Change	Statistic	p-value	FDR
IL-1β	38.8 ± 29.4	20 ± 0	0.52	t = 1.56	0.18	1
IL-6	148.7 ± 253.0	16.01 ± 0	0.11	t = 1.28	0.26	1
IL-4	0.63 ± 0.57	0.4 ± 0	0.63	t = 1	0.36	1
IL-10	55 (55, 67.6)	55 (55, 339.3)	1.00	Wilcoxon W = 15	0.60	1
MCP-1	1302 ± 833	1473 ± 326	1.13	t = -0.47	0.65	1
IL-13	10.4 ± 5.5	9.1 ± 5.6	0.87	t = 0.43	0.68	1
TNF-α	21403 ± 8974	20026 ± 4521	0.93	t = 0.34	0.75	1
IL-2	145.5 ± 130.7	145.6 ± 108.7	1.00	t = -0.001	0.99	1
∗IFN-γ	5.3 ± 0	5.3 ± 0	1.00	= NA	1.00	1
IL-1α	20 (20, 135.4)	20 (20, 1276.0)	1.00	Wilcoxon W = 18	1.00	1

Data expressed as either mean ± S.D or as the median with minimum and maximum values.

∗ IFN-γ excluded because of zero variance.

## 3 Discussion

Our preclinical results provide evidence that pregnancy is characterized by enhanced expression of sialylated IAV cellular entry factors. Furthermore, we show increased presence of pDCs in the nasal epithelium and a robust Type I IFN-specific response after stimulation with a TLR7/8 agonist during pregnancy, suggesting both an increased state of innate immune surveillance and the possibility of uncontrolled inflammation, respectively.

Severity of both pandemic and seasonal IAV infection in pregnancy is attributed to a variety of factors, including, but not limited to, the hormonal environment and physiological changes in the immune, cardiovascular, and respiratory systems [9, 27, 28]. Though hormonal changes of pregnancy are known to increase systemic sialylation [20, 21, 23, 24], whether they do so in the respiratory tract remains unclear. This is a critical knowledge gap because the host factors for both avian and human IAV cell entry are sialylated glycoproteins — sialic acid [SA] α-2,3, and α-2,6-linked glycans, respectively. These sialoglycans show highly specific binding with either human or avian IAV [29, 30, 31], are expressed throughout the respiratory tract with species- and anatomical region-specific differences [19, 29, 32, 33], and facilitate viral entry by binding to IAV hemagglutinins [34]. Here, using both immunoblotting and lectin-based flow cytometry, we confirmed for the first time, the increased expression of these sialoglycan receptors during pregnancy. However, to validate our findings, future experiments that involve mass spectrometric quantification of sialic acid residues are recommended. Though we investigated IAV receptors only in epithelial cells, we recognize that immune cells such as pDCs express them as well. However, we believe that the epithelial cells are the major source in the reproductive age group because the frequencies of epithelial cells outstrip the frequencies of immune cell populations in the nasal epithelium by a factor of approximately 10:1 [35]. Increased availability of these receptors could potentially promote enhanced entry of IAV into respiratory epithelial cells raising the possibility that pregnant women could be more susceptible to IAV infection, in addition to suffering from severe disease.

An increase in the population of pDCs in the nasal epithelium of pregnant rats was another intriguing finding, contrary to current evidence pointing to a decrease in the frequency of pDCs in the systemic circulation during pregnancy [36, 37, 38]. Because pDCs are potent producers of Type I IFNs [39], we evaluated this with intranasal instillation of resiquimod, a potent TLR-7/8 agonist [40, 41] that mimics IAV infection. We noted an approximately 25% increase in IFN-α concentration in the nasal epithelium of pregnant *vs*. non-pregnant rats; though the cellular origin of IFN-α in our experiments remains unresolved, we believe that pDCs are the likely drivers of this response because of their disproportionately high contribution to IFN-α production. These findings may appear contradictory to our hypothesis but are supported by evidence for increased activation of pDCs from pregnant women infected *in vitro* with IAV [11]. While a robust Type I IFN antiviral response may be considered protective, it is often a two-edged sword. Excessive and uncontrolled Type I IFN signaling, for example, is frequently associated with severe immunopathology during influenza A [42, 43, 44]. In addition, we had previously shown that the expression of viral nucleic acid sensors (*Rig-1*, *Tlr7*, *MyD88*, and *Irf7*) was significantly enhanced in the nasal epithelium of pregnant rats [26]. Because a sublethal IAV load can paradoxically use the physiological inflammation mediated by TLR-7 pathway to enhance viral replication [45], it is possible that such infection in pregnancy can leverage these upregulated pathways, enhance viral replication, and worsen clinical outcomes. In addition, pDC function is influenced by pregnancy-associated hormones [46, 47, 48], therefore, inter-individual differences in plasma levels of these hormones in pregnancy have the potential to alter the immune response. Overall, our findings of enhanced innate immune surveillance in the nasal epithelium of pregnancy are broadly consistent with upregulated Type I IFN-mediated innate immune function in pregnancy [49].

Given the robust Type I IFN production in the nasal epithelium during pregnancy, it is unclear why the clinical outcomes of pandemic influenza A and coronavirus disease-19 (COVID-19), caused by severe acute respiratory syndrome coronavirus 2 (SARS-CoV-2) infection, are strikingly different in pregnant women. Though current data suggest that symptomatic pregnant patients with SARS-CoV-2 infection are more likely to need admission to the intensive care unit and mechanical ventilation [50, 51, 52], a vast majority of patients remain asymptomatic compared to non-pregnant patients with COVID-19 [53, 54, 55, 56, 57]. Furthermore, the mortality rates (approximately 1–1.5%) for COVID-19 during pregnancy are similar to those observed in age- and sex-matched populations [58, 59, 60, 61, 62], in contrast to the high mortality and morbidity rates observed with seasonal and pandemic influenza A [18, 28, 63]. Based on our previous report showing that factors necessary for the cellular entry of SARS-CoV-2 are downregulated in the nasal epithelium of pregnant rats [26], we believe that the contrasting clinical presentations could partly stem from differential nasal epithelial cell entry of these viruses resulting in varying magnitudes of Type 1 IFN production and subsequent immunopathology.

Our preclinical work has important implications for future research and points to critical lines of clinical investigation that need to be pursued: (i) are pregnant women susceptible to IAV infection in addition to severe disease? and (ii) are the immune changes in pregnancy site-specific? The first question can be answered mechanistically by assessing (i) the nasal transcriptome and proteome in pregnant subjects to determine the expression and regulation of IAV receptors across the gestational period, (ii) evaluating the viral load in pregnant *vs*. age-, sex-, and symptom-matched non-pregnant women, and (iii) performing *in vitro* viral entry and replication studies in respiratory epithelial cells isolated from pregnant women. Epidemiologically, it could be addressed by assessing the overall rate of influenza-like illness in pregnant compared to age-matched women during a distinct time epoch. Another important implication of our study is that the concept of generalized immunological indolence during pregnancy is probably erroneous; our results showing a markedly robust innate immune response in the nasal epithelium suggest that severe IAV infection in pregnant women is likely due to excessive inflammation rather than a deficient immune response. Therefore, high-resolution studies of the nasal immune cell populations during pregnancy are urgently warranted.

Major strengths of our work include the innovative use of lectin-based flow cytometry to quantify the sialoglycans in the nasal epithelium and the novel use of intranasal resiquimod to assess Type I IFN production. Nevertheless, our study has a few limitations. First, the causal link between increased tissue concentration of sex steroids during pregnancy and upregulation of IAV receptors remains to be determined. However, limited evidence from ovariectomized mice shows that estradiol induces ST6GAL1 expression and increases systemic α-2,6-linked sialylation [64], suggesting that this is a possibility. Similarly, given the increase in systemic sialylation across the trimesters of pregnancy [20, 22, 24], it is necessary to perform studies to determine if expression of IAV receptors varies across the different trimesters to confer altered susceptibility to infection. Second, because of the lack of a BSL-3 facility, we were unable to perform viral tropism studies to confirm if pregnant rats were more susceptible to severe IAV infection. This limitation, however, is offset partially by previously published studies in mice demonstrating that the pulmonary viral load is approximately 8-fold higher with an amplified inflammatory response after nasal IAV inoculation in pregnant compared to their non-pregnant counterparts [13, 65, 66, 67]. Third, our studies were conducted in pregnant rats at term gestation because (i) pregnancy-related hormonal levels typically plateau during this period, and (ii) influenza is more severe at this stage of pregnancy [68]. Therefore, we recommend against extrapolating our findings to earlier stages of pregnancy. Fourth, the absence of a negative control for lectin-based flow cytometry can be considered a limitation. However, previous work has shown that lectin-glycan interactions are highly specific [69] and are mediated by multivalent binding to a cluster of specific carbohydrate recognition domains [70]. Fifth, is the concern that the pDCs identified in the nasal epithelium could be derived from the circulation and may not be tissue-resident. However, we minimized that possibility by thoroughly rinsing the freshly dissected nasal epithelium to remove the capillary blood. Furthermore, presence of tissue-resident pDCs in the nasal epithelium of healthy human subjects has been reported previously [71]. Sixth, the efficacy of intranasal resiquimod may be questioned; however, the Type I IFN concentration was 2-3-fold higher after resiquimod administration compared to control providing confidence that resiquimod was highly effective in stimulating the TLR-7 pathway. Finally, despite the primordial importance of the nasal epithelium in host-pathogen interaction, it is premature to speculate about the clinical course of IAV infection, because it is more likely to be influenced by innate immune response/inflammation in the lung and changes in systemic immunity. Invasive lower respiratory tract studies are, therefore, necessary to comprehensively understand the pathogenesis of influenza infections in pregnancy.

Collectively, our research highlights two important findings regarding the nasal epithelium during pregnancy: (i) upregulation of IAV receptors, and (ii) a Type I IFN-biased environment that appears to be driven by increased pDCs. Exaggerated IAV immunopathology in pregnancy is, therefore, likely due to excessive, rather than a deficient, Type I IFN-mediated immune response. Considering the novelty and clinical relevance of these findings, a comprehensive high-resolution map of the nasal epithelial transcriptome and innate immune cell populations is urgently needed during pregnancy to fully understand host-respiratory pathogen interaction in this unique demographic.

## 4 Materials and methods

### 4.1 Animals

All experiments were conducted after appropriate institutional approval (protocol ID:19–1071; Washington University School of Medicine, St. Louis, MO) and comply with the ARRIVE (Animals in Research: Reporting *In Vivo* Experiments) guidelines. Because of the pregnancy-specific nature of the research question, only pregnant and non-pregnant adult female rats were used in the study (CD® Sprague Dawley IGS strain, Charles River Laboratories). Pregnant dams were singly housed while non-pregnant females were housed in pairs at 22 °C under a 12 ​h–12 ​h light-dark cycle. We chose gestational day 20 (GD20) to perform these experiments in pregnant rats because (i) the gestational age reflects the third trimester of human pregnancy when IAV infection is notably severe [18, 72], and (ii) to avoid the possibility of inducing birth from immune provocation studies (a possibility if performed close to term gestation at GD22). Both groups had access to water and standard chow *ad libitum*. Group sizes (n = 6–9) were determined based on the effect sizes observed during our previous study [26]. Experiments reported here were conducted between Aug 2020–Jun 2021 and all efforts were made to minimize the number of animals used for the study.

### 4.2 Dissection of the nasal epithelium

Nasal epithelial tissue was dissected from euthanized term pregnant rat at GD20 and age-matched non-pregnant rats according to the protocol described by Dunston et al. with modifications [73]. Briefly, the head of the euthanized rat was decapitated with a sharp blade, the scalp was peeled, and the lower jaw was disarticulated. Under light microscope, the nasal bones were meticulously dissected, starting at the ventral surface of the vomer bone followed by the entire dorsal area. The nasal epithelium was carefully stripped from the bony attachments, snap frozen, and stored at -80 °C.

### 4.3 Pregnancy-associated hormone assays

Because of lack of information on the nasal tissue concentration of hormones during pregnancy, we assayed 17-β estradiol (LS-F55510, LifeSpan BioSciences, Inc.) and progesterone (LS-F39173, LifeSpan BioSciences, Inc.) in nasal epithelial lysates according to manufacturers’ instructions. Briefly, lysates were prepared from approximately 50 mg of nasal epithelial tissue in 1x PBS (0.02 mol/L, pH 7.2) using a Bullet Blender homogenizer (Next Advance, Inc., Troy, NY). The lysates were subsequently centrifuged at 16000 g at 4 °C for 10 min, supernatants were collected, and their protein concentrations determined using BCA Protein Assay Kit (Thermo Fisher Scientific, Inc.). For estradiol and progesterone assays, approximately 10 μg and 150 μg equivalent protein from each sample were applied to the designated well(s), respectively. To better understand the systemic hormonal environment during pregnancy, we performed plasma estradiol and progesterone assays. Prior to collection of the nasal epithelium, 1 mL of blood was directly aspirated from the left ventricle using a 21G needle, mixed in a BD Vacutainer EDTA tube, and subsequently centrifuged at 700 g × 10 min. Approximately 500 μL of plasma was stored at -80 °C and thawed immediately prior to the hormone assays. All experiments were performed in duplicate, and the absorbance was read with Tecan Infinite M200 PRO (Tecan Group Ltd., Switzerland) at 450 nm. Hormone concentrations were determined by comparison with predetermined standards and reported as either pg/mg of protein or pg/mL.

### 4.4 Western blot for IAV receptors

Lysates for western blot were prepared from approximately 50 mg of nasal epithelium in RIPA buffer (50 mM Tris HCl pH 7.5, 150 mM NaCl, 2 mM EDTA, 1% NP40, 0.1% SDS) with protease and phosphatase inhibitor cocktail (ThermoFisher Scientific Inc.), using a Bullet Blender homogenizer (model-storm 24, Next Advance, Inc., Troy, NY). The lysates were subsequently centrifuged at 16000 g at 4 °C for 10 min. Protein concentrations of the collected supernatants were determined using BCA Protein Assay Kit (ThermoFisher Scientific, Inc.). Approximately 30 μg of protein was subjected to gel electrophoresis and transferred to membrane using Bolt western blot reagents from ThermoFisher Scientific Inc (bolt 4–12% Bis Tris gel, #NW04125; bolt sample reducing agent, #B0009; bolt LDS sample buffer, #B0007; iBlot2 dry blotting system). For ST3GAL4 staining (α-2, 3- linked sialylated glycans), iBind Automated Western Systems (iBind Flex; ThermoFisher Scientific Inc.) using their proprietary iBind Flex Fluorescent Detection (FD) Solution Kit (SLF2019), according to manufacturers' instructions. The anti-ST3GAL4 antibody (#MBS9133580, MyBioSource Inc.) and the secondary antibody (anti-Rabbit AF680; #A21084, ThermoFisher Scientific Inc.) were used at 1:250 and at 1:1000 dilution, respectively, and exposed to Odyssey Fc Imaging System (LI-COR Biosciences, Inc.). The images were processed with Image studio ver 5.2 (LI-COR) for densitometric quantification. For ST6GAL1 staining (α-2,6-linked sialylated glycans), the membrane was blocked with TBST buffer containing 5% milk for 1 h at room temperature on a shaker. Following a brief wash with TBST buffer, the membrane was immunoblotted overnight at 4 °C on a shaker with anti-ST6GAL1 (#MBS3216285, MyBioSource Inc.) at a dilution of 1:500 in TBST buffer containing 5% milk. HRP-conjugated secondary antibody (anti-Rab IgG, #7074, Cell Signaling Technology) was used at a dilution of 1:1000 for 1 h at room temperature on a shaker. Immunoblot was developed with Western ECL substrate (#1705060, Bio-Rad Laboratories Inc.) for 5 min at room temperature and exposed to Odyssey Fc Imaging System as described above. For protein normalization, the membranes were stripped with Western Reprobe Plus (#786-307, G-Biosciences, St. Louis, MO), according to manufacturers’ instructions and probed with HRP conjugated β-Actin (sc-47778HRP, Santa Cruz Biotechnology, Inc.) at a dilution of 1:1000 for 1 h at room temperature on a shaker and developed as mentioned above. Rat intestine was used as positive control.

### 4.5 Lectin-based flow cytometry for IAV receptors

Nasal epithelial tissues were harvested from pregnant (GD20) and age-matched nonpregnant female rats for flow cytometry experiments. Single cell suspensions were prepared using the following protocol. Tissues were minced and digested with RPMI media supplemented with FCS and Collagenase-IV for 40 min at 37 °C. Cells were filtered through 70-μm filters to remove clumps, washed twice using FACS buffer (2% FCS-PBS) and stained with required antibodies and appropriate controls. Cells were analyzed by flow cytometry using a BD FACS Canto II instrument (BD Biosciences, San Jose, CA) and data were analyzed using FlowJo (BD Biosciences Inc.). The following antibodies were used: FITC∗SNA-I (Sambucus nigra agglutinin-I; #F-6802-1, EY Laboratories Inc., San Mateo, CA), Biotin∗MAA (Maackia amurensis agglutinin; #BA-7801-2, EY Laboratories Inc., San Mateo, CA), Brilliant Violet 421∗Streptavidin (#405226, BioLegend, Inc.), mAb-EpCAM (GZ-1) (#ab187276, Abcam plc., Cambridge, MA) and APC Goat anti-mouse IgG (#405308, BioLegend, Inc.). Dead cells were excluded from analysis by staining with LIVE/DEAD Fixable Blue Dead Cell stain kit (#L23105, ThermoFisher Scientific Inc.). Live, singlet, EpCAM^+^ and CD45^-^ cells were considered as epithelial cells, upon which the expression of SNA-I and MAA were scored.

### 4.6 Flow cytometric identification of innate immune cell types in the nasal epithelium

Single cell suspensions were prepared and stained for flow cytometry during the same set of experiments described above. The following fluorescent-labeled antibodies were used: APC/cy7∗anti-rat CD45 (#202216, BD Biosciences), Brilliant Violet 421∗anti-rat CD3 clone 1F4 (RV0) (#563948, BD Biosciences), APC∗anti-rat CD11b clone WTS (RV0) (#562102 BD Biosciences), FITC∗ anti-rat RT1Dab (MHCII) (#205405, BioLegend, Inc.), PerCP/cy 5.5∗ anti-rat CD4 (#201519, BioLegends, Inc.), and PE∗ anti-rat CD161 (#205604, BioLegends Inc.). Dead cells were excluded by staining with LIVE/DEAD Fixable Blue Dead Cell stain kit (#L23105, ThermoFisher Scientific, Inc.) during analysis. Live lymphocyte sized cells were stained with CD45 and CD3 to identify T cells. Further the T cells were divided into T helper and cytotoxic T cells based on CD4 and CD8 expression, respectively. CD3-, CD11b-, CD4+ and MHCII + cells were identified as plasmacytoid dendritic cells (pDCs). CD3-and CD161 + cells were identified as natural killer (NK) cells (gating strategy is presented as Supplementary Material).

### 4.7 Instillation of intranasal resiquimod

We had previously reported an increase in Toll-like receptor (TLR)-7 gene expression in the nasal epithelium of pregnant rats [26]. To determine whether this was associated with an increase in interferon response, we administered resiquimod (R848), a synthetic TLR-7/8 agonist, intranasally. We chose resiquimod because R848 has been shown to produce innate immune responses in the respiratory tract that are broadly comparable to those observed with live IAV infection [74]. Briefly, mildly anesthetized (isoflurane 2% for 3–4 min) dams and non-pregnant rats were suspended in the erect position using a custom-designed apparatus and resiquimod solution (approximately 150 μL of 2 mg/ml dissolved in 10% DMSO, MedChemExpress Inc.), was quickly delivered drop-by-drop with the help of a 200 μL pipette to both nostrils equally followed by returning the rats to their respective cages. Following 2 h of exposure to resiquimod, the animals were euthanized, and nasal epithelium was collected as described above for interferon and cytokine assays. The choice of 2-hour timepoint was guided by previous work in mice and humans showing maximal Type I interferon response at 2–3 h after R-848 [75, 76, 77].

### 4.8 Type I IFN response

The concentrations of IFN-α and IFN-β were measured using the Rat Interferon Alpha ELISA kit (#MBS4500053, MyBioSource Inc.) and the Rat Interferon Beta ELISA kit (#MBS4500062, MyBioSource, Inc.), respectively, according to manufacturer's instructions. In brief, nasal epithelial lysates were prepared from approximately 50 mg tissue in 1x PBS (0.02 mol/L, pH 7.2) using a Bullet Blender homogenizer (Next Advance, Inc., Troy). The lysates then were centrifuged at 16000 g at 4 °C for 10 min and supernatants were collected. Protein concentrations were determined using BCA Protein Assay Kit (Thermo Fisher Scientific, Inc.). For IFN-α and IFN-β assay, approximately 150 ug equivalent proteins from each sample were applied to the designated well(s). All experiments were performed in duplicate, and the absorbance was read with Tecan Infinite M200 PRO (Tecan Group Ltd., Switzerland) at 450 nm. Interferon concentrations were determined by comparison with predetermined standards and reported as pg/mg of protein.

### 4.9 Cytokine response

Quantitative measurement of 10 cytokines (IFN-γ, IL-1α, IL-1β, IL-2, IL-4, IL-6, IL-10, IL-13, MCP-1, and TNFα) were assayed using the Quantibody® Rat Inflammation Array Q1 kit (# QAR–INF–1, RayBiotech Life Inc., GA), according to manufacturers’ instructions. The Quantibody array is a sandwich ELISA-based quantitative array platform (glass slide-based) that accurately determines the concentration of multiple cytokines simultaneously. In brief, nasal epithelial lysates were prepared, and protein concentrations determined as described above. Approximately 125 μg equivalent protein from each sample were added to glass slides precoated with capture antibodies (multiple cytokine-specific) that capture the target cytokines. A second biotin-labeled antibody that recognize a different epitope of the target cytokine was then added and visualized through the addition of the streptavidin-conjugated Cy3 equivalent dye using a laser scanner (GenePix® Professional 4200A, Molecular Devices LLC.). Data extraction (GAL file) was done using microarray analysis software (GenePix® Pro Microarray Analysis Software, Molecular Devices LLC.). For cytokine quantification, the array specific cytokine standards were assayed in each array simultaneously to generate a standard curve for each cytokine. By comparing signals from unknown samples to the standard curve, the cytokine concentration in the samples were determined.

### 4.10 Statistical analysis

Data outliers were eliminated using ROUT (robust regression and outlier analysis) with Q set to 10% and normality of residuals was assessed with D'Agostino-Pearson omnibus test. Normally and non-normally distributed data (except array data) were analyzed with Welch's t-test and Mann-Whitney U test, respectively. Interferon release data were analyzed with 2-way ANOVA. Quantibody array data were summarized either as mean ± SD, or median with minimum and maximum values across the groups. The fold change between groups was calculated as the ratio of the mean or the median. If the cytokines met or did not meet normality criteria across the group, the significance of expression difference was evaluated with either t-test or Wilcoxon-Mann-Whitney test, respectively. Non-array data were analyzed with Prism 9 for macOS (version 9.1.2; GraphPad Software Inc.) and presented as mean ± SEM; p ≤ 0.05 was accorded statistical significance. Computation and analyses of the Quantibody cytokine array data were performed in the array-specific Q-Analyzer Tool (QAR–INF–1-SW, RayBiotech Life Inc.) using the company-provided biostatistics & bioinformatics service (R programming language V3.6.3). Cytokines with FDR <0.05 were considered as differentially expressed.

## 5 Data sharing statement

The equipment needed to perform this research are commercially available and non-proprietary. All data needed to evaluate the accuracy of conclusions are submitted with the paper. Data on the baseline interferon status in the nasal epithelium of rats are available upon request from the authors.

## Declarations

### Author contribution statement

Arvind Palanisamy: Conceived and designed the experiments; Analyzed and interpreted the data; Wrote the paper.

Tusar Giri: Performed the experiments; Analyzed and interpreted the data; Wrote the paper.

Santosh Panda: Performed the experiments; Contributed reagents, materials, analysis tools.

Jeannie Kelly and Carlo Pancaro: Analyzed and interpreted data; Wrote the paper.

### Funding statement

This research did not receive any specific grant from funding agencies in the public, commercial, or not-for-profit sectors.

### Data availability statement

Data included in article/supplementary material/referenced in article.

### Declaration of interests statement

The authors declare no conflict of interest.

### Additional information

No additional information is available for this paper.

## Appendix A Supplementary data

Heliyon Supplementary File_Final Heliyon Supplementary File_Final
