# Supplementary material for: Upregulated influenza A viral entry factors and enhanced interferon-alpha response in the nasal epithelium of pregnant rats
Source: Heliyon. 2022 May 11;8(5):e09407. doi: 10.1016/j.heliyon.2022.e09407 (PMC9111991; doi:10.1016/j.heliyon.2022.e09407)
Supplement: Heliyon Supplementary File_Final [file mmc1.pdf]

Supplementary File

Upregulated influenza A viral entry factors and enhanced interferon-alpha response in the nasal epithelium of pregnant rats

Uncropped western blot files for Fig. 2A

ST3GAL4

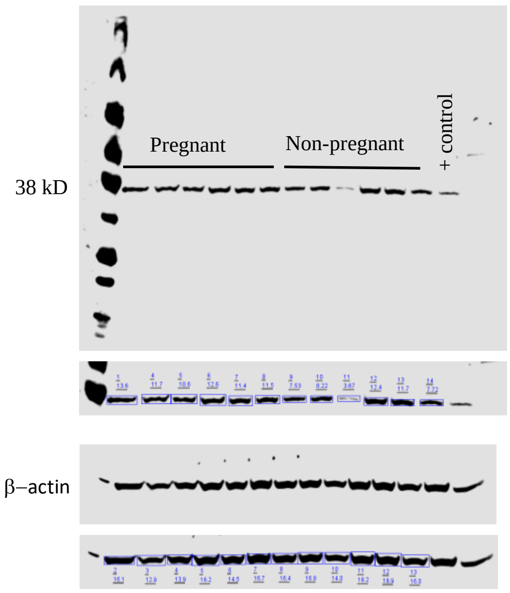

ST6GAL1

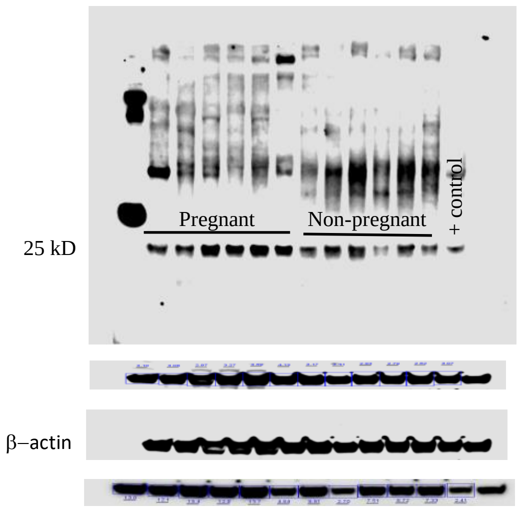

Please note the reversed order in the uncropped files with pregnant followed by non-pregnant samples. Rat intestine was used as positive control.

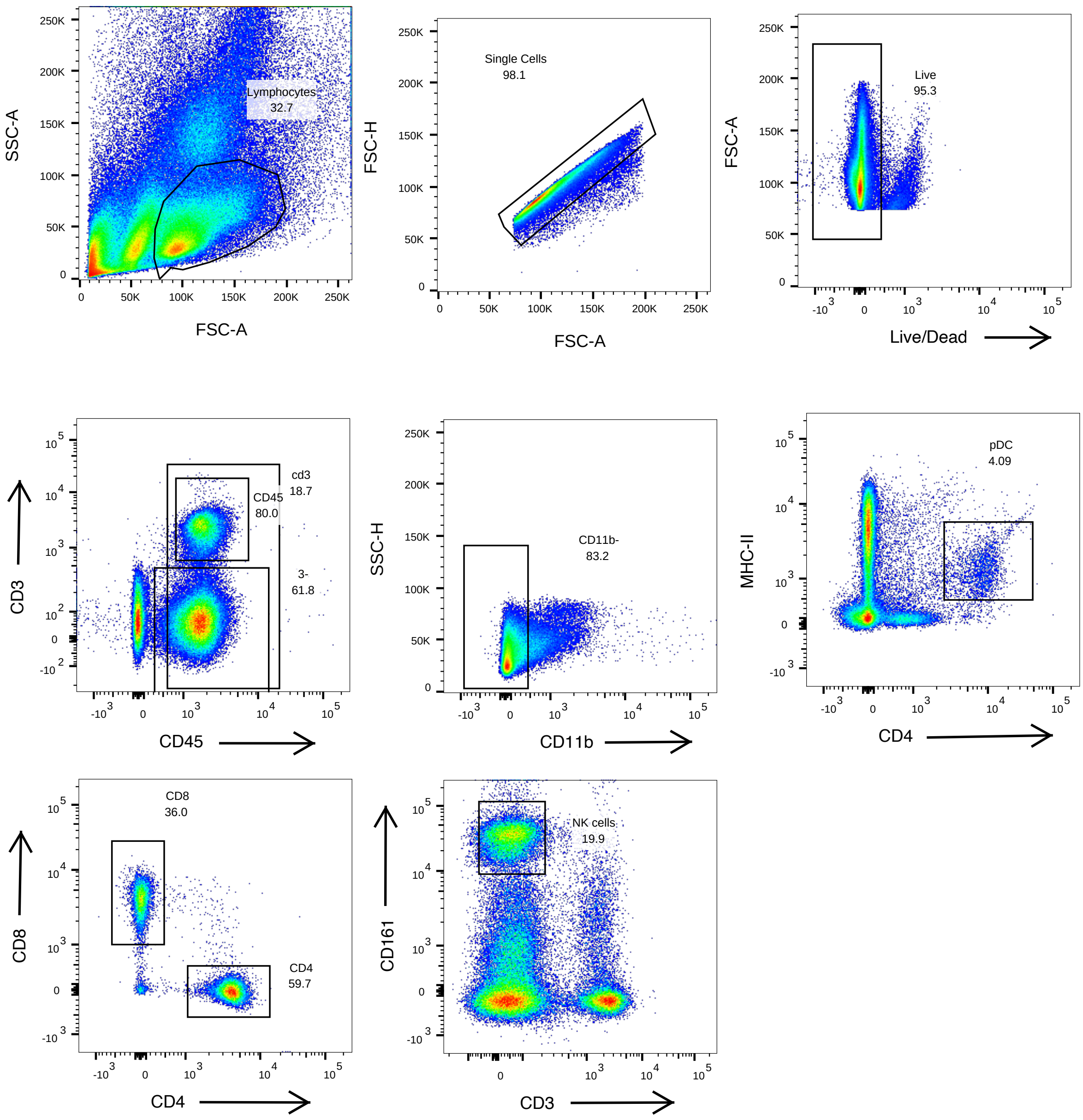

Lymphocytes
32.7
SSC-A
FSC-A
Single Cells
98.1
FSC-H
FSC-A
Live
95.3
FSC-A
Live/Dead
cd3
18.7
CD45
80.0
3-
61.8
CD3
CD45
CD11b-
83.2
SSC-H
CD11b
pDC
4.09
MHC-II
CD4
CD8
36.0
CD4
59.7
CD8
CD4
NK cells
19.9
CD161
CD3
